# Is the Headache in Patients with Vestibular Migraine Attenuated by Vestibular Rehabilitation?

**DOI:** 10.3389/fneur.2017.00124

**Published:** 2017-04-03

**Authors:** Nagisa Sugaya, Miki Arai, Fumiyuki Goto

**Affiliations:** ^1^Unit of Public Health and Preventive Medicine, School of Medicine, Yokohama City University, Yokohama, Japan; ^2^Department of Otolaryngology, National Hospital Organization Tokyo Medical Center, Tokyo, Japan

**Keywords:** vestibular diseases, migraine disorders, rehabilitation, treatment, vestibular rehabilitation, headache impact, dizziness handicap

## Abstract

**Background:**

Vestibular rehabilitation is the most effective treatment for dizziness due to vestibular dysfunction. Given the biological relationship between vestibular symptoms and headache, headache in patients with vestibular migraine (VM) could be improved by vestibular rehabilitation that leads to the improvement of dizziness. This study aimed to compare the effects of vestibular rehabilitation on headache and other outcomes relating to dizziness, and the psychological factors in patients with VM patients, patients with dizziness and tension-type headache, and patients without headache.

**Methods:**

Our participants included 251 patients with dizziness comprising 28 patients with VM, 79 patients with tension-type headache, and 144 patients without headache. Participants were hospitalized for 5 days and taught to conduct a vestibular rehabilitation program. They were assessed using the Dizziness Handicap Inventory (DHI), Headache Impact Test (HIT-6), Hospital Anxiety and Depression Scale (HADS), and Somatosensory Catastrophizing Scale (SSCS) and underwent center of gravity fluctuation measurement as an objective dizziness severity index before, 1 month after, and 4 months after their hospitalization.

**Results:**

The VM and tension-type headache groups demonstrated a significant improvement in the HIT-6 score with improvement of the DHI, HADS, SSCS, and a part of the objective dizziness index that also shown in patients without headache following vestibular rehabilitation. The change in HIT-6 during rehabilitation in the VM group was positively correlated with changes in the DHI and anxiety in the HADS. Changes in the HIT-6 in tension-type headache group positively correlated with changes in anxiety and SSCS.

**Conclusion:**

Vestibular rehabilitation contributed to improvement of headache both in patients with VM and patients with dizziness and tension-type headache, in addition to improvement of dizziness and psychological factors. Improvement in dizziness following vestibular rehabilitation could be associated with the improvement of headache more prominently in VM compared with comorbid tension-type headache.

## Introduction

The association between vestibular symptoms, including dizziness and vertigo, and headache has been reported in several studies (1, 2). While various terms have been used to describe the combination of vestibular dysfunction and migraine, “vestibular migraine” (VM) is the accepted name for vestibular symptoms that are causally related to migraine (3). The diagnostic criteria for VM are described in the International Classification of Headache Disorder (ICHD), third edition, beta version (4, 5).

Some studies have proposed that the mechanism underlying vestibular dysfunction relating to migraine is a parallel activation of vestibular and cranial nociceptive pathways (6–9). Nociceptive and vestibular afferents with neurochemical similarities, including expression of serotonin, capsaicin, and purinergic receptors (6, 10) converge in brainstem structures such as the parabrachial nucleus, raphe nuclei, and locus coeruleus, and these structures play an important role in modulating the sensitivity of pain pathways (3). Thus, vestibular symptoms may have a biologically close relation to headache.

There is now a widespread consensus that an exercise-based therapy known as “vestibular rehabilitation” or “balance retraining” is the most effective treatment for dizziness due to vestibular dysfunction (11). Our previous research also reported that vestibular rehabilitation contributes to the improvement of perceived handicaps due to dizziness, catastrophization of bodily sensation, and emotional distress (12). Given the biological relationship between vestibular symptoms and headache as described previously, headache in patients with VM could be improved by vestibular rehabilitation that leads to the improvement of dizziness. Regarding the effect of vestibular rehabilitation in VM, a previous study reported that both patients with VM and those with vestibular impairment exhibited significant improvement of vestibular symptoms following rehabilitation (13). However, the effect of vestibular rehabilitation on headache has not been demonstrated, and it is not clear whether there is any relationship between the improvement of headache and that of dizziness during rehabilitation.

Therefore, the present study aimed to compare the effects of vestibular rehabilitation on headache and other outcomes relating to dizziness, and psychological factors between patients with VM, patients with dizziness and tension-type headache, and patients with dizziness and without headache and to investigate the association between improvements in headache and other outcomes.

## Materials and Methods

### Participants

The participants of the present study were patients with a chief complaint of dizziness who visited the department of otorhinolaryngology in the National Tokyo Medical Center between February 2015 and August 2016. Participants reported that they felt persistent dizziness even after conventional treatment, which included (1) drug therapy with betahistine (14) 36 mg daily for the first 2–4 weeks, (2) life style counseling to conduct daily exercise including walking, and (3) sleeping sufficiently and stress reduction. We recruited participants for this study from this pool of patients if (1) the patient was ≥20 years old, (2) the dizziness had persisted for at least 3 months despite conventional therapy in the outpatient clinic, (3) the patient wished to have intensive, inpatient therapy for the persistent dizziness, and (4) the patient was literate. Our exclusion criteria were (1) a diagnosis of dizziness due to cerebrovascular disorder, (2) medical contraindications for making the necessary head movements during vestibular rehabilitation (for example, severe cervical disorder), (3) serious comorbidity (for instance, a life-threatening condition, severe cognitive impairment, or severe psychiatric disorder), (4) central nervous system disease, or (5) bilateral vestibular deficit. There were 470 patients who met the listed requirements. Migraine and tension-type headache were strictly defined, and the other types of primary headaches (e.g., cluster headache and trigeminal autonomic cephalalgias) and secondary headaches (e.g., headache attributed to trauma or injury to the head and/or neck) were carefully ruled out. The diagnosis of VM, as well as that of tension-type headache, was based on the ICHD, third edition, beta version (4).

This study was approved by the ethical committee of the National Tokyo Medical Center (R12-009) and has, therefore, been performed in accordance with the ethical standards laid down in the 1964 Declaration of Helsinki and its later amendments.

### Measures

#### Headache Impact Test (HIT-6)

Headache Impact Test (15) measures the impact of headaches on a patient’s life and consists of 6 items. The HIT-6 was the main outcome in this study. Response options include (with corresponding relative weights in parentheses): never (6), rarely (8), sometimes (10), very often (11), and always (13). Four groups have been derived to aid in the interpretation of HIT-6 scores: scores ≤49 represent little or no impact, scores between 50 and 55 represent some impact, scores between 56 and 59 represent substantial impact, and scores ≥60 indicate severe impact (16).

#### Frequency of Headache

Frequency of headache, used as an adjunct to the HIT-6, was rated by 1 item on a 9-point scale as follows: never or almost never (0), less than once a month (1), at least once a month (2), once a week (3), two to three times a week (4), four to six times a week (5), once a day (6), more than once a day (7), and always (8).

#### Dizziness Handicap Inventory (DHI)

The DHI (17, 18) is a standard questionnaire that quantitatively evaluates the degree of handicap in the daily lives of patients with vestibular disorders and consists of 25 questions. The total score ranges from 0 (no disability) to 100 (severe disability).

#### Frequency of Dizziness

Frequency of dizziness, used as an adjunct to the DHI, was rated on a 9-point scale as follows: never or almost never (0), less than once a month (1), at least once a month (2), once a week (3), two to three times a week (4), four to six times a week (5), once a day (6), more than once a day (7), and always (8).

#### Hospital Anxiety and Depression Scale (HADS)

The HADS (19) is a self-report questionnaire consisting of 14 questions on a 4-point scale, consisting of an anxiety subscale with 7 items and a depression subscale with 7 items. This psychometric instrument was chosen because all its items refer solely to an emotional state and do not consider somatic symptoms.

#### Somatosensory Catastrophizing Scale

The Somatosensory Catastrophizing Scale (SSCS) (20) is a 27-item, self-report measure that evaluates catastrophization of bodily sensation using a 5-point scale ranging from “not at all true” to “extremely true,” where higher scores indicate greater perceived catastrophization of bodily sensation.

#### The Gravity Center Fluctuation Measurement

The gravity center fluctuation measurement for objective assessment of the severity of dizziness was performed using a stabilometer (G-5000, Anima Corp., Tokyo), and provided the total length of path (LNG) and environmental area (ENV) during eye-opening/closing.

### The Intervention

Patients were hospitalized for 5 days in groups of 8–10 individuals; the groups were then taught how to perform the 30-min vestibular rehabilitation program by themselves (21). The program comprised repeated training of the vestibulo-ocular (VOR) and vestibulo-spinal reflexes (VSR). The VOR training included seven exercises: (1) quick horizontal eye movement, (2) quick vertical eye movement, (3) eye tracking horizontal direction, (4) eye tracking vertical direction, (5) horizontal head shaking with gazing fixed target, (6) vertical head shaking with gazing fixed target, and (7) oblique head tilting with gazing fixed target. Each eye or head movement was repeated 20 times. The VSR training consisted of eight static and five dynamic exercises. The eight static exercises were (1) standing up and sitting down with eyes open, three times; (2) standing up and sitting down with eyes closed, three times; (3) standing with eyes closed and feet open for 20 s; (4) standing with eyes closed and feet closed for 20 s; (5) standing with tandem gait with right foot in front for 20 s; (6) standing with tandem gait with left foot in front for 20 s; (7) one leg stand on the right foot for 20 s; and (8) one leg stand on the left foot for 20 s. The five dynamic programs were (1) 180° turn to the left, three times; (2) 180° turn to the right, three times; (3) walking with tandem gait for 10 m; (4) walking with horizontal head shaking for 10 m; and (5) walking with vertical head shaking for 10 m. During education, patients performed these exercises three times a day under the supervision of a physician. After 5 days, all patients had learned how to perform the exercises. The patients were then instructed to continue performing the vestibular rehabilitation program three times a day after discharge.

### Procedure

After the participants had provided written, informed consent, they were evaluated on the day of hospitalization (time 1), as well as 1 and 4 months afterward (time 2 and time 3), using the questionnaires mentioned previously. Static posturography was also conducted. All drugs that could affectdizziness, including vestibular suppressants, were removed soon after the introduction of vestibular rehabilitation.

### Statistical Analysis

The data analysis was performed using the SPSS 22.0 software (SPSS, Chicago, IL, USA). The participants were divided into three groups: (1) patients with VM complaining of a current headache and an HIT-6 score ≥50 (VM group), (2) patients with chronic and episodic tension-type headache and a HIT-6 score ≥50 (tension-type headache group), and (3) patients without a headache and an HIT-6 score ≤49 (non-headache group). The non-headache group included patients diagnosed with VM without a current headache. A two-way repeated measures analysis of variance (ANOVA) was performed to analyze the effects of group and time on all outcomes. The *t*-test was used for group comparisons of age. Correlation analysis (Pearson’s correlation coefficient) was used to examine the relationship between changes in outcomes during rehabilitation. The significance level was set at less than 5%.

## Results

### Characteristics of the Participants

**Figure 1:**
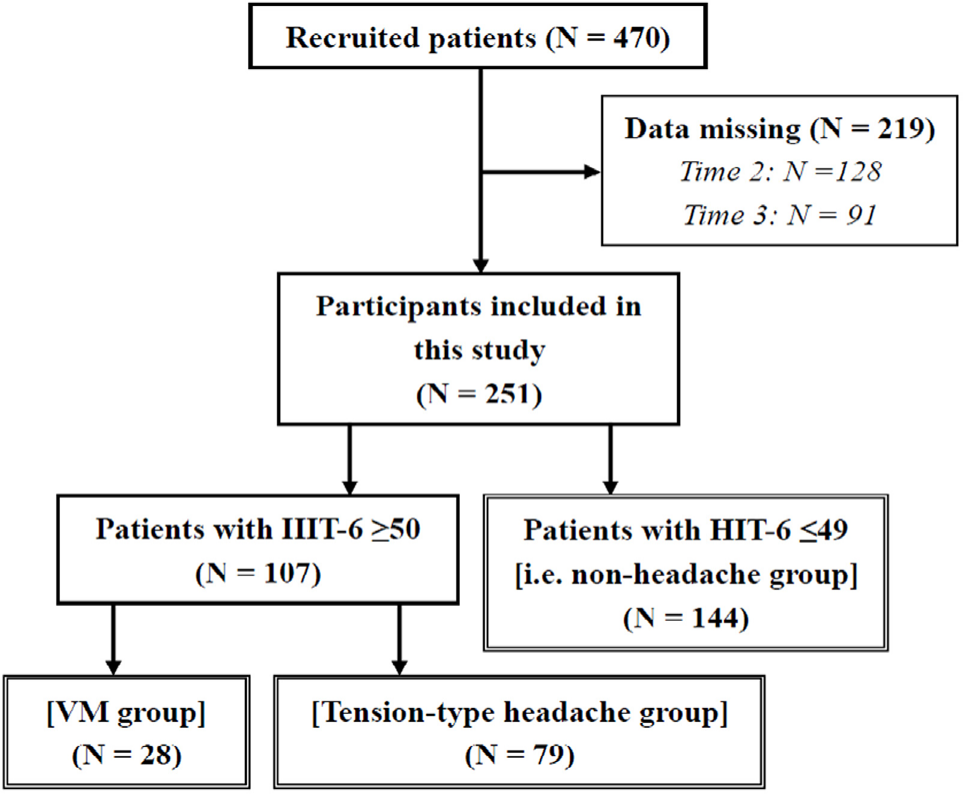
**Number of participants in this study**. VM, vestibular migraine; HIT-6, Headache Impact Test.

There were 470 patients who met the requirements listed. We further excluded those who had data missing on the HIT-6 at any time 1–3 (*n* = 219); thus, 251 patients (56 male and 195 female patients, mean age = 62.64 ± 16.40 years) remained in the analysis. The participants with an HIT-6 score ≥ 50 at time 1 included 28 patients in the VM group (all patients were female) and 79 patients in the tension-type headache group (16 males and 63 females). Our participants did not include any patients with other types of primary or secondary headaches. The other 144 patients with an HIT-6 score ≤49 at time 1 were assigned to the non-headache group (40 males and 104 females) (Figure 1). The non-headache group included four patients diagnosed with VM without a current headache. Some patients did not provide any data regarding the frequency of headache and dizziness (valid data: *N* = 237), DHI responses (valid data: *N* = 242), HADS responses (valid data: *N* = 249), SSCS responses (valid data: *N* = 239), LNG during eye opening (valid data: *N* = 238) and closing (valid data: *N* = 234), and ENV during eye opening (valid data: *N* = 237) and closing (valid data: *N* = 233). In cases where the participant fell, their center of gravity fluctuation measurement was treated as missing data. Table 1 shows the diagnoses of the participants, according to a history taken during their initial visit. The ANOVA showed a significant difference in age between groups (VM < tension-type headache < non-headache; Table 2).

**Table 1:** **Diagnoses of the participants**.

Diagnosis	Total	VM group	Tension-type headache group	Non-headache group
VM with current headache	28	28	0	0
VM without current headache	4	0	0	4
Unilateral vestibulopathy	49	0	16	33
Vestibular neuritis	31	0	15	16
BPPV (HC or PC)	19	0	3	16
Ménière’s disease	25	0	13	12
Sudden deafness with vertigo	33	0	13	20
Recurrent vestibulopathy	9	0	4	5
Psychogenic dizziness	31	0	10	21
Post-traumatic brain injury	4	0	2	2
Presbystasis	9	0	1	8
Other	9	0	2	7
Total	251	28	79	144

*VM, vestibular migraine; BPPV, benign paroxysmal positional vertigo; HC, horizontal canal type; PC, posterior canal type*.

**Table 2:** **Comparisons of frequency of dizziness, emotional distress, somatic catastrophizing, and center of gravity fluctuation measurements between groups and time points (two-way repeated measures analysis of variance)**.

		VM group	Tension-type headache group	Non-headache group	Main effect of time		Main effect of group		Interaction	
				
		Mean ± SD	Mean ± SD	Mean ± SD	*F*	*p*	*F*	*p*	*F*	*p*
Age		47.68 ± 18.18	58.80 ± 16.38	67.66 ± 13.58	–	–	24.41	<0.0001	–	–

Frequency of dizziness	Time 1	5.36 ± 2.36	5.01 ± 2.75	4.61 ± 3.09	39.67	<0.0001	0.39	NS	0.85	NS
	Time 2	3.43 ± 2.30	3.90 ± 2.56	3.47 ± 3.02						
	Time 3	2.96 ± 2.28	3.07 ± 2.66	3.04 ± 3.07						

HADS-A	Time 1	8.86 ± 4.40	10.53 ± 4.90	6.64 ± 4.17	34.12	<0.0001	23.87	<0.0001	1.10	NS
	Time 2	6.93 ± 4.98	7.57 ± 4.58	4.65 ± 3.54						
	Time 3	7.07 ± 4.31	7.51 ± 4.38	4.58 ± 3.70						

HADS-D	Time 1	7.57 ± 3.68	9.66 ± 4.06	6.32 ± 3.79	32.64	<0.0001	21.00	<0.0001	0.52	NS
	Time 2	6.04 ± 3.70	7.65 ± 4.14	4.86 ± 3.68						
	Time 3	5.54 ± 3.95	7.25 ± 4.10	4.54 ± 3.37						

SSCS	Time 1	91.48 ± 20.86	88.10 ± 21.33	77.63 ± 22.01	66.80	<0.0001	6.16	0.002	2.11	NS
	Time 2	70.93 ± 24.24	75.15 ± 20.40	65.22 ± 20.50						
	Time 3	70.26 ± 23.18	72.51 ± 23.10	66.35 ± 22.54						

LNG eye opening	Time 1	101.58 ± 39.87	108.83 ± 46.06	112.57 ± 46.91	1.92	NS	0.74	NS	0.10	NS
	Time 2	98.65 ± 40.43	113.89 ± 113.92	112.24 ± 88.31						
	Time 3	89.14 ± 28.74	100.80 ± 44.92	103.47 ± 54.29						

LNG eye closing	Time 1	131.33 ± 52.91	159.72 ± 106.87	155.68 ± 84.31	8.45	0.001	0.68	NS	0.77	NS
	Time 2	137.98 ± 103.38	143.12 ± 76.20	146.48 ± 83.45						
	Time 3	115.62 ± 36.97	132.20 ± 77.54	136.92 ± 78.37						

ENV eye opening	Time 1	5.92 ± 4.11	6.22 ± 5.04	5.68 ± 4.39	0.07	NS	0.36	NS	0.23	NS
	Time 2	6.46 ± 8.59	6.00 ± 4.59	5.22 ± 6.08						
	Time 3	5.86 ± 4.43	5.97 ± 6.35	5.50 ± 8.92						

ENV eye closing	Time 1	10.03 ± 10.38	9.72 ± 11.70	8.64 ± 9.95	4.40	0.02	0.61	NS	1.41	NS
	Time 2	12.25 ± 29.30	8.36 ± 13.80	7.06 ± 10.17						
	Time 3	6.64 ± 4.75	7.22 ± 7.72	7.04 ± 9.09						

*VM, vestibular migraine; HADS-A and D, Hospital Anxiety and Depression Scale—Anxiety scale and Depression scale; SSCS, Somatosensory Catastrophizing Scale; LNG, total length of path; ENV, environmental area*.

*Results of post hoc test—age: VM < tension-type headache < non-headache; frequency of dizziness: time 1 > 2 > 3; HADS-A: time 1 > 2 and 3; VM and tension-type headache > non-headache; HADS-D: time 1 > 2 and 3; tension-type headache > VM and non-headache; SSCS: time 1 > 2 and 3; tension-type headache > non-headache; LNG during eye closing: time 1 and 2 > 3; ENV during eye closing: time 1 > 3*.

### Two-Way ANOVA Results

**Figure 2:**
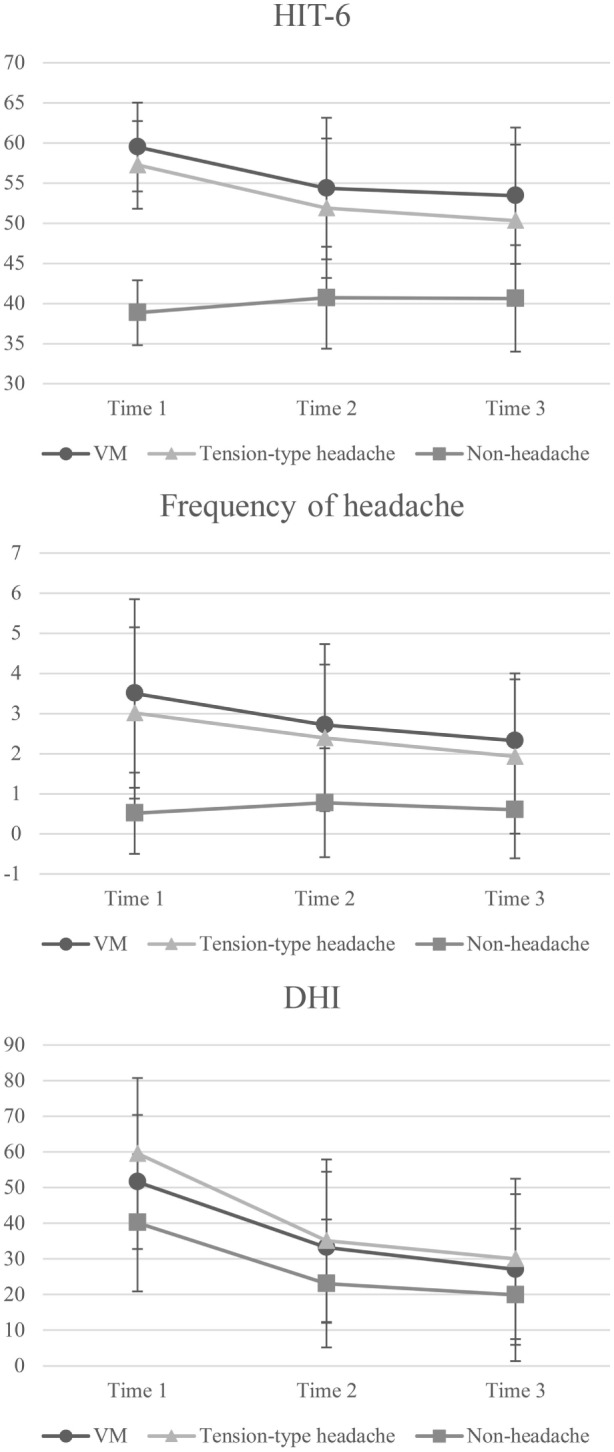
**Two-way repeated measures ANOVA of headache and dizziness scores for comparisons between groups and time points**. Error bars represent the SD. VM, vestibular migraine; HIT-6, Headache Impact Test; DHI, Dizziness Handicap Inventory. HIT-6—main effect of time: *F* = 22.43, *p* < 0.0001; main effect of group: *F* = 212.24, *p* < 0.0001; interaction: *F* = 23.09, *p* < 0.0001. *Post hoc* test: VM and tension-type headache groups > non-headache group at time 1–3; time 1 > time 2 and 3 in the VM and tension-type headache groups; time 1 < time 2 and 3 in the non-headache group. Frequency of headache—main effect of time: *F* = 14.40, *p* < 0.0001; main effect of group: *F* = 71.77, *p* < 0.0001; interaction: *F* = 7.94, *p* < 0.0001. *Post hoc* test: VM and tension-type headache groups > non-headache group at time 1–3; time 1 > 3 in VM group; time 1 > 2 and 3 in the tension-type headache group. DHI—main effect of time: *F* = 164.46, *p* < 0.0001; main effect of group: *F* = 17.37, *p* < 0.0001; interaction: *F* = 3.68, *p* = 0.01. *Post hoc* test: VM and tension-type headache groups > non-headache group at time 1; tension-type headache group > non-headache group at time 2–3; time 1 > 2 and 3 in the VM group; time 1 > 2 > 3 in the tension-type headache and non-headache groups.

Regarding the HIT-6 score (Figure 2; Table 2), there was a significant interaction between group and time. The *post hoc* test showed that the VM and tension-type headache groups had a higher score than the non-headache group at all time points and that the scores at tie 2 and 3 were lower than that at time 1 in the VM and tension-type headache groups. However, the scores at time 2 and 3 were higher than that at time 1 in the non-headache group.

In the frequency of headache score (Figure 2), there was a significant interaction between group and time. *Post hoc* testing indicated that the VM and tension-type headache groups had higher scores than the non-headache group at all time points and that the scores at 2 and 3 were lower than that at time 1 in both the VM and tension-type groups.

In terms of DHI score (Figure 2), there was a significant interaction between group and time. *Post hoc* testing indicated that the VM and tension-type headache groups had higher scores than the non-headache group at time 1, while only tension-type headache had a higher score than the non-headache group at time 2–3. In addition, the scores at time 2 and 3 were lower than those at time 1 in the VM group while the scores in tension-type headache and non-headache were time 1 > time 2 > time 3.

In the frequency of dizziness (Table 2), significant main effect of time was found; however, there was no significant interaction between group and time and main effect of group. *Post hoc* testing showed that the scores were time 1 > time 2 > time 3.

In the HADS-A and HADS-D results (Table 2), significant main effects of group and time were found, but there was no significant interaction between group and time. *Post hoc* testing for HADS-A showed that the scores at time 2 and 3 were lower than that at time 1 and that the scores in the VM and tension-type headache groups were higher than those in the non-headache group. *Post hoc* testing for HADS-D showed that the scores at time 2 and 3 were lower than that at time 2 and that the scores in the tension-type headache group were higher than those in the VM and non-headache groups.

Regarding the SSCS scores (Table 2), there were significant main effects of group and time. *Post hoc* testing indicated that the scores at time 2 and 3 were lower than those at time 1 and that the score in the tension-type headache group was higher than that in the non-headache group.

Regarding the center of gravity fluctuation measurements (Table 2), LNG and ENV during eye closing showed significant main effects of time, but there was no significant interaction between group and time. *Post hoc* testing indicated that LNG at time 2 and 3 was smaller than that at time 1 and that ENV at time 3 was smaller than that at time 1. In LNG and ENV during eye opening, there was no significant interaction or main effect.

### Results of Correlation Analysis

As the HIT-6 score, the main outcome in this study, significantly increased from time 1 to time 2, the change in all variables improved by the rehabilitation was calculated by subtracting the score at time 2 from that at time 1 (Table 3). In the VM group, change in the HIT-6 score was significantly correlated with changes in DHI and HADS-A. In the tension-type headache group, changes in HIT-6 significantly correlated with changes in HADS-A and SSCS.

**Table 3:** **Correlation between changes from time 1 to time 2**.

			DHI	HADS-A	HADS-D	SSCS	LNG eye closing	ENV eye closing
VM group	HIT-6	*r*	0.51	0.51	0.32	0.37	0.01	−0.06
		*p*	0.01	0.005	0.09	0.06	0.95	0.78

Tension-type headache group	HIT-6	*r*	0.10	0.23	0.14	0.30	−0.16	−0.13
		*p*	0.40	0.048	0.22	0.01	0.16	0.27

*VM, vestibular migraine; HIT-6, Headache Impact Test; DHI, Dizziness Handicap Inventory; HADS-A and D, Hospital Anxiety and Depression Scale—Anxiety scale and Depression scale; SSCS, Somatosensory Catastrophizing Scale; LNG, total length of path; ENV, environmental area*.

## Discussion

Regarding the results of the two-way repeated measures ANOVA, patients with dizziness and current headache (i.e., the VM and tension-type headache groups) demonstrated a significant improvement in headache with coincident improvement of their dizziness and psychological variables following vestibular rehabilitation. On the other hand, although patients without headache demonstrated a significant improvement in dizziness and their psychological variables following rehabilitation, their headache HIT-6 score was increased at 1 month after discharge; however; their scores at all time points had no clinical importance (the HIT-6 scores <50). The reason of this result in patients without headache could be caused by increased awareness of symptoms other than dizziness due to the improvement of dizziness. Vitkovic et al. (13) reported that the subjective scores, including dizziness and psychological aspects, at baseline for patients with VM reflected a heightened perception of symptoms compared with patients with vestibular impairment and that this difference was maintained even after 6 months. However, the patients with VM received the same degree of benefit from rehabilitation as patients with vestibular impairment in both subjective and objective measures of the severity of dizziness. Our results demonstrated similar trends to those found in the above previous study regarding subjective scores, including the HIT-6 and objective scores.

In the results of the correlation analysis, change of headache impact from baseline to 1 month after discharge in the VM group significantly and positively correlated with changes of perceived handicap due to dizziness and anxiety. Additionally, the change of headache impact in the tension-type headache group significantly and positively correlated with changes of anxiety and catastrophization of bodily sensation. The results of the correlation analysis indicated the possibility that the improvement in dizziness following vestibular rehabilitation contributed to the improvement of headache more prominently in the VM group compared with the tension-type headache group. We presumed that the biological relationship between dizziness and headache previously described could mediate the relationship between the improvement in dizziness and headache. In the tension-type headache group, the improvement in headache could be mainly related to the improvement in psychological aspects, including maladaptive cognition and anxiety, with a beneficial change in dizziness following vestibular rehabilitation.

Although patients with dizziness and headache, including the VM and tension-type headache groups, demonstrated a significant improvement in HIT-6 score 1 month after starting vestibular rehabilitation, their scores at 1 and 4 months after discharge remained above 50, a fact that is clinically pertinent. Another intervention specific to headache may be needed in addition to vestibular rehabilitation for patients with dizziness and headache. Moreover, various factors mediating the biological link between dizziness and headache are involved in the formation of anxiety responses (3). The combination of vestibular rehabilitation and intervention for emotional distress (e.g., stress management) could contribute to the improvement of both dizziness and headache more effectively. Particularly in the tension-type headache group, intervention for their strong maladaptive cognition could be effective in improving whole physical symptoms, including dizziness and headache.

Although there was a significant difference in age between groups (VM group < tension-type headache group < non-headache group), we could not perform an analysis of covariance controlling for age as a covariate because the regression lines of the dependent variables and age were not parallel. However, age has previously been reported to be related to headache (22, 23). Further investigation in a larger sample is needed to compare the effects of vestibular rehabilitation by the presence of VM or other headache types and by age group.

In current specialized medical services, physicians specializing in dizziness have provided medical care focusing on dizziness symptoms for patients with dizziness and headache, while physicians specializing in headache have provided medical care focusing on headache for the same patient group. Thus, VM or headache in patients with dizziness has not been investigated sufficiently. To our knowledge, this is the first study to investigate both headache and dizziness symptoms in patients with VM and patients with dizziness and other types of headache. Vestibular rehabilitation does not need any specialized equipment to be performed, and patients can practice the rehabilitation by themselves in their own home. Given our results that vestibular rehabilitation may improve the severity of migraine in the VM group, we suggest that vestibular rehabilitation could be a beneficial intervention in clinical practice for migraine in the case of comorbidity of dizziness with migraine.

The present study had several limitations. First, we only verbally confirmed the participants’ compliance with the rehabilitation program, and we did not ask them to keep a record of their rehabilitation. Second, the number of VM patients was small. In future, we should conduct a similar study in a larger sample and try to compare between sexes and between age groups or apply appropriate regression analyses. Third, we assigned the participants to each group based on the HIT-6 score at baseline. This method could cause a regression to the mean and affect the result such that the HIT-6 score in non-headache group was increased 1 month after discharge although their scores at all time points had no clinical importance. In future research, we should assign the participants to each group using longitudinal data about headache severity before initiating vestibular rehabilitation. Fourth, this study lacked a control group. The effect of vestibular rehabilitation on headache is worth investigating using a randomized controlled protocol and obtaining more convincing evidence.

## Conclusion

Vestibular rehabilitation contributed to the improvement of headache both in patients with VM and patients with dizziness and tension-type headache, in addition to the improvement of dizziness and psychological factors. The improvement in dizziness following vestibular rehabilitation contributed to the improvement in headache more prominently in the VM group in comparison with the tension-type headache group.

## Author Contributions

NS wrote the manuscript. MA collected the data. FG organized the research and checked the manuscript.

## Conflict of Interest Statement

The authors declare that the research was conducted in the absence of any commercial or financial relationships that could be construed as a potential conflict of interest.
